# Tonsillar cytotoxic CD4 T cells are involved in the control of EBV primary infection in children

**DOI:** 10.1038/s41598-024-52666-4

**Published:** 2024-01-25

**Authors:** María Eugenia Amarillo, Agustina Moyano, Natalia Ferressini Gerpe, Elena De Matteo, Maria Victoria Preciado, Paola Chabay

**Affiliations:** 1 Molecular Biology Laboratory, Pathology Division, Multidisciplinary Institute for Investigation in Pediatric Pathologies (IMIPP), CONICET-GCBA, Ricardo Gutierrez Children′s Hospital, Gallo 1330, C1425EFD Ciudad Autónoma de Buenos Aires, Argentina; 2 Pathology Division, Ricardo Gutierrez Children′s Hospital, C1425EFD Ciudad Autónoma de Buenos Aires, Argentina

**Keywords:** Microbiology, Pathogenesis

## Abstract

CD4 T cells play a key role in Epstein Barr virus (EBV) infection, by modulating latent antigen expression, and exhibiting cytotoxic and regulatory properties. Our aim was to evaluate the presence of Granzyme B (GZMB) and Foxp3 CD4 T cells at different EBV infection status and latency profiles. We examined CD4, GZMB, Foxp3, IL10, TGF-β, CD4-GZMB and CD4-Foxp3 expression at the tonsils of pediatric patients with different infective status and EBV latency profiles. CD4+, GZMB+, Foxp3+, CD4-GZMB+ and CD4-Foxp3+ cell counts were higher at the interfollicular region. Higher expression of CD4-GZMB was found in primary infected patients compared to healthy carriers. In patients that expressed latency III antigens, we demonstrated lower CD4+, CD4-GZMB+, CD4-Foxp3+ expression; a negative correlation between the immunoregulatory cytokine IL-10+ and GZMB+ as well as a positive correlation of IL-10+ and CD4+. In patients expressing the lytic protein BMRF1, a positive correlation of TGF-β+ with CD4-GZMB+ and CD4-Foxp3+ was observed. Our findings indicate that CD4-GZMB+ cells are involved in the restriction of primary EBV infection in pediatric patients, which could partially explain the lack of symptoms, whereas both CD4-GZMB+ and CD4-Foxp3+ cells could be involved in the modulation of latency.

## Introduction

The Epstein–Barr virus (EBV), alternatively referred to as human gammaherpesvirus 4, has successfully infected over 90% of the global adult population^1^. Like all herpesviruses, the EBV establishes both lytic and latent cycles within the host, in which it is latently maintained for life as an episome. Mostly in children EBV infection is asymptomatic, but in adults it can manifest as infectious mononucleosis (IM). EBV is transmitted from carriers through saliva and once it enters the oropharyngeal region the virus gains access to one of the main sites of viral infection and reactivation, the tonsil, where the main target cell for EBV resides, the B lymphocyte. Upon primary infection, the nine viral latency proteins as well as non-coding RNAs are expressed in infected B lymphocytes, defining a latency profile III. During the physiological maturation of B lymphocytes, the expression of viral latency proteins is silenced, leading to a shift to a latency II profile, where only EBV nuclear antigen 1 (EBNA1), Latent membrane protein 1 (LMP1), Latent membrane protein 2A (LMP2A), EBV-encoded RNAs (EBERs) and BamHI A-encoded transcripts (BARTs) are expressed. Subsequently, the virus completely silences the expression of all viral latency proteins (latency profile 0) except for B lymphocytes in active division, that only express EBNA1 (latency profile I)^2^.

Notably, EBV is estimated to contribute to approximately 1% of all human malignancies. The relatively infrequent occurrence of virus-induced diseases in healthy infected individuals could be attributed to the presence of a robust immune response against EBV, particularly mediated by EBV-specific CD8 and CD4 T cells. In adult patients with IM, a large expansion of activated CD8 T cells was described^3^, particularly targeting EBV lytic epitopes. On the other hand, during IM, the expansion of CD4 T cells is small, and their response is mainly against EBV latency antigens. The role of CD4 T cells is essential for immune protection against viruses^4^. In the context of EBV infection, CD4 T cells have been shown to modulate virus latent antigen expression in vitro, providing relevant evidence for understanding virus infection at different virus infection statuses^5^. For several years now, the role of cytotoxic CD4 T cells in the control of viral infections and in tumor immunity has been recognized. However, these cells exhibit various cytotoxic mechanisms, similar to those seen in cytotoxic CD8 T cells and natural killer (NK) cells, such as the production of cytotoxic granules containing granzyme B (GZMB). Concerning immunity against EBV, multiple studies have documented the presence of EBV-specific cytotoxic CD4 T cells^6^.

CD4 regulatory T cells (Tregs) are crucial for maintaining immunological self-tolerance and homeostasis. It is well-established that these cells specifically express the transcription factor Foxp3. Tregs employ various mechanisms to suppress immune response, including the release of anti-inflammatory cytokines such as IL-10 and TGF-β^7^. Previous studies have reported elevated Foxp3+ Tregs in patients with EBV-positive Hodgkin lymphoma (HL)^8^. Furthermore, it has been described that EBV influences the tumor microenvironment of HL and strategically contributes to immune evasion by increasing the tumor microenvironment with Treg cells and the concomitant presence of immunosuppressive cytokines in adult HL^9^.

In Argentina, EBV infection typically presents as subclinical, with approximately 90% of individuals seroconverting by the age of 3. Moreover, a statistical association has been observed between EBV and B-cell lymphoma in patients under the age of 10, implying a potential link between early EBV seroconversion and an elevated risk of B-cell lymphoma development^10^. In addition, our group previously described in pediatric patients with HL a cytotoxic and inflammatory environment characterized by GZMB+ counterbalanced by a regulatory milieu with Foxp3+ cells as a marker of Tregs in EBV+ cases^11^. Furthermore, also in pediatric HL from Argentina, a positive correlation was proved between CD4+ and GZMB+ cells count, suggesting a role of cytotoxic CD4 T cells^12^.

Therefore, as little is known about the response of local cytotoxic and regulatory CD4 T cells in EBV primary and persistent infection in pediatric patients, from developed as well as undeveloped populations, our aim was to evaluate the presence of GZMB+ and Foxp3+ CD4 T cells at different EBV infections statuses as well as in relation to different latency profiles in an undeveloped population.

## Results

### Latency profiles and EBV infection status

According to the presence of EBV antibodies, our cohort was classified into four groups (Supplementary Table S1). Of the 50 children analyzed, 16 (32%) were primary infected (PI), 9 (18%) undergoing reactivation (R), 21 (42%) healthy carriers (HC) and 4 (8%) non-infected (NI). The expression of viral latent antigens studied by EBERs ISH and IHC (Supplementary Table S2). 13 patients expressing Latency III antigens, 14 Latency II,12 Latency I and 7 Latency 0. In addition, the expression of the lytic antigen BMRF1 was detected in 10 cases. Age distribution of EBV infection status is indicated in Supplementary Table S3.

### CD4, GZMB and Foxp3 single expression in relation to EBV

In order to explore whether there is a balance between GZMB and Foxp3 in the early steps of EBV infection, or during viral persistence, Foxp3+ and GZMB+ expression in the tonsils was evaluated in a cohort of pediatric patients in the context of different EBV infection states.

First, we studied whether there was a correlation between Foxp3+ and GZMB+ in all patients, considering each EBV infection status and each expression of EBV latency profile independently; but this analysis showed no correlation in any of these conditions (*p* > 0.05).

In addition, we evaluated whether there was a correlation between CD4 and GZMB at different infection status and latency profiles. There was no moderate or high correlation between GZMB+ and CD4+ cells neither in the whole cohort, nor EBV infection status or latent antigen expression (*p* > 0.05).

**Figure 1:**
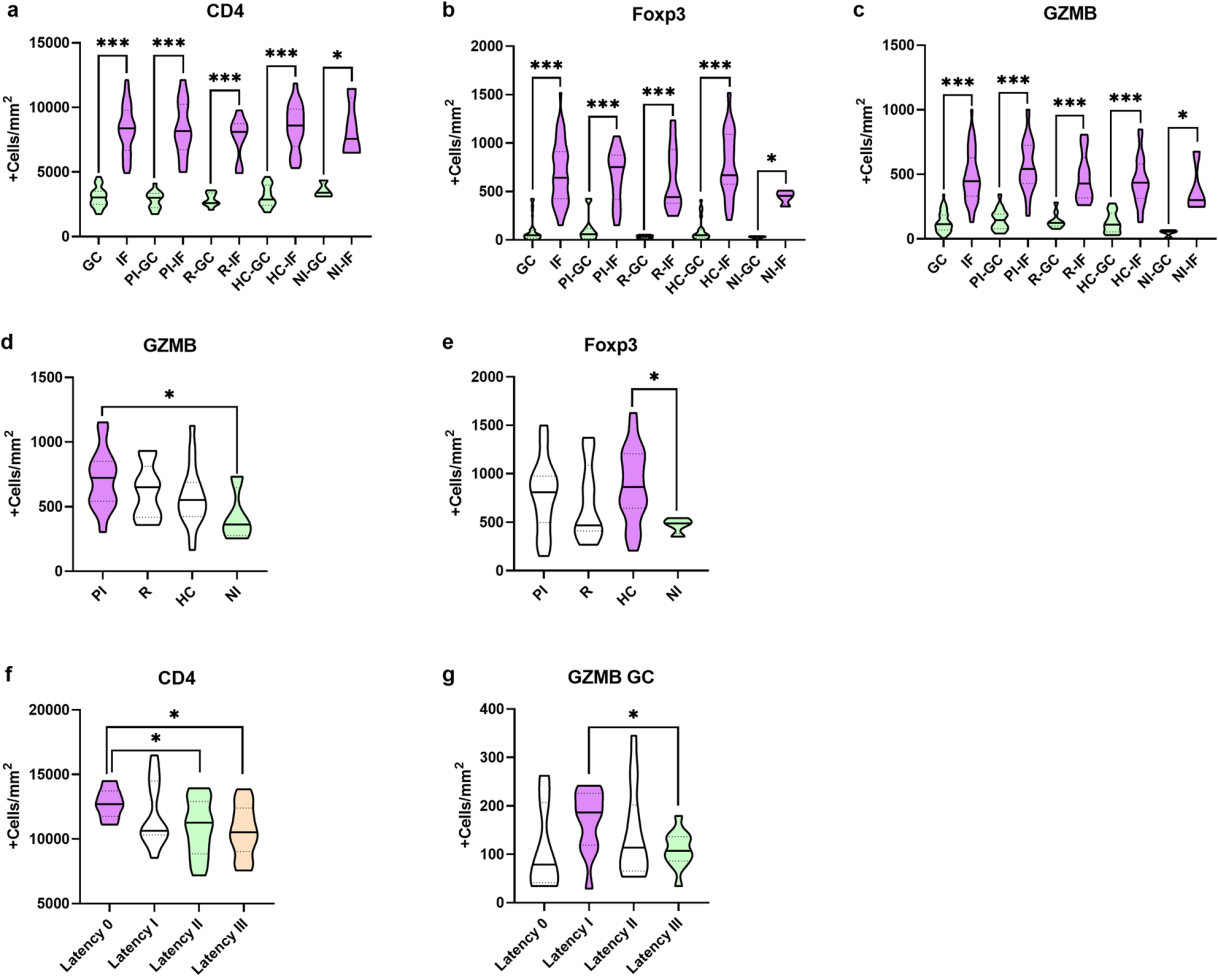
Histological distribution, patients classified according to EBV infection status and EBV latency profile with respect to CD4+ , Foxp3+ and GZMB+ expression. Histological distribution of CD4+ (**a**), Foxp3+ (**b**) and GZMB+ (**c**) expression in the germinal center (GC) and interfollicular region (IF) in the entire cohort, primary infected (PI) patients, patients undergoing reactivation (R), healthy carriers (HC) and non-infected patients (NI). Patients classified according to EBV infection status with respect to GZMB+ (**d**) and Foxp3+ (**e**) expression in primary infected patients (PI), patients undergoing reactivation (R), healthy carriers (HC) and non-infected patients (NI). Patients classified according to EBV latency profile with respect to CD4+ (**f**) and GZMB+ at the GC (**g**) in patients with latency 0, I, II and III. ANOVA or Kruskal Wallis test, when appropriate, with Bonferroni or Dunn’s test for multiple comparison, respectively, where applied when the 4 infection groups or latency profiles were compared. Horizontal bars indicate M–W or T-test, when appropriate, comparison between 2 infection or latency profile groups. *p < 0.05 **p < 0.01 ***p < 0.001.

Concerning the histological distribution, CD4+, GZMB+ and Foxp3+ cell counts were higher at the IF region compared to the GC (p˂0.0001) considering the whole cohort and each infection status analyzed separately (p˂0.05) (Fig. 1a–c respectively). There was no significant difference in CD4+ cells count among the EBV infection statuses (*p* > 0.05) (Supplementary Table S4).

A lower count for GZMB+ was observed in NI compared to PI patients (*p* = 0.0483) (Fig. 1d). Furthermore, regarding the GC region, GZMB+ cells were statistically lower in NI patients with respect to PI and R patients (*p* = 0.0149 and *p* = 0.0028 respectively). A lower Foxp3+ cell count was observed in NI patients respect to HC patients (*p* = 0.0456) (Fig. 1e) (Supplementary Table S4)**.**

When the patients were stratified according to the latency antigens expression in tonsils, the CD4+ count was higher in those patients with latency profile 0 compared to those with latency profile II and III (*p* = 0.0442 and *p* = 0.0197, respectively) (Fig. 1f). This result was preserved at the IF region (*p* = 0.0451 and *p* = 0.0088 respectively), but not at the GC (*p* > 0.05). In addition, there were no differences in the GZMB+ cell count in the whole tonsil among the different EBV latency profiles in this cohort (*p* > 0.05). Particularly at the GC, GZMB+ cell count was significantly higher in patients with latency profile I compared to patients with latency profile III (*p* = 0.0122) (Fig. 1g), while no differences were proved among the different latency profiles in the IF region (*p* > 0.05). Concerning the Foxp3+ count, there was no significant difference when patients were compared according to the EBV latency profiles (*p* > 0.05) (Supplementary Table S5). In addition, when lytic antigen expression was evaluated, no statistical differences were observed for CD4+, Foxp3+ and GZMB+ mean cells counts in cases that express lytic BMRF1 antigen compared to negative cases (*p* > 0.05) (Supplementary Table S6).

### CD4-GZMB and CD4-Foxp3 double expression in relation to EBV

**Figure 2:**
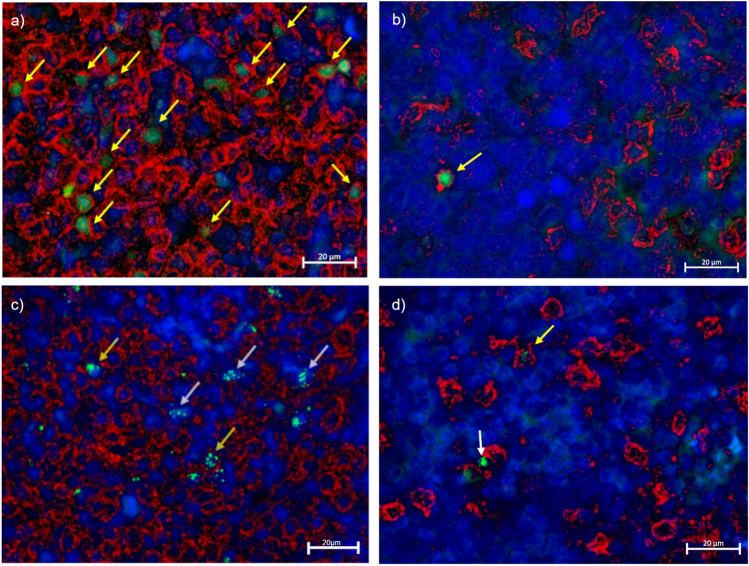
Double staining for CD4-GZMB+ and CD4-Foxp3+ in pediatric patients´tonsils. Double stain for Foxp3+ (Green) and CD4+ (Red) at 1000X (**a**) at the interfollicular (IF) region of tonsil and (**b**) at the GC. Double stain for GZMB+ (green) and CD4+ (Red) at 1000X (**c**) at the IF region of tonsil and (**d**) at the GC. Yellow arrow: Double stain for CD4-Foxp3+ and CD4-GZMB+ . White arrow: single stain for GZMB+ . Each image includes a nuclear counterstain Hoechst (blue).

In order to study the contribution of CD4 T cells to Foxp3+ and GZMB+ expression, we analyzed CD4-GZMB+ and CD4-Foxp3+ T cell counts according to the histological distribution, distinct infection status and latency profiles (Fig. 2a–d).

**Figure 3:**
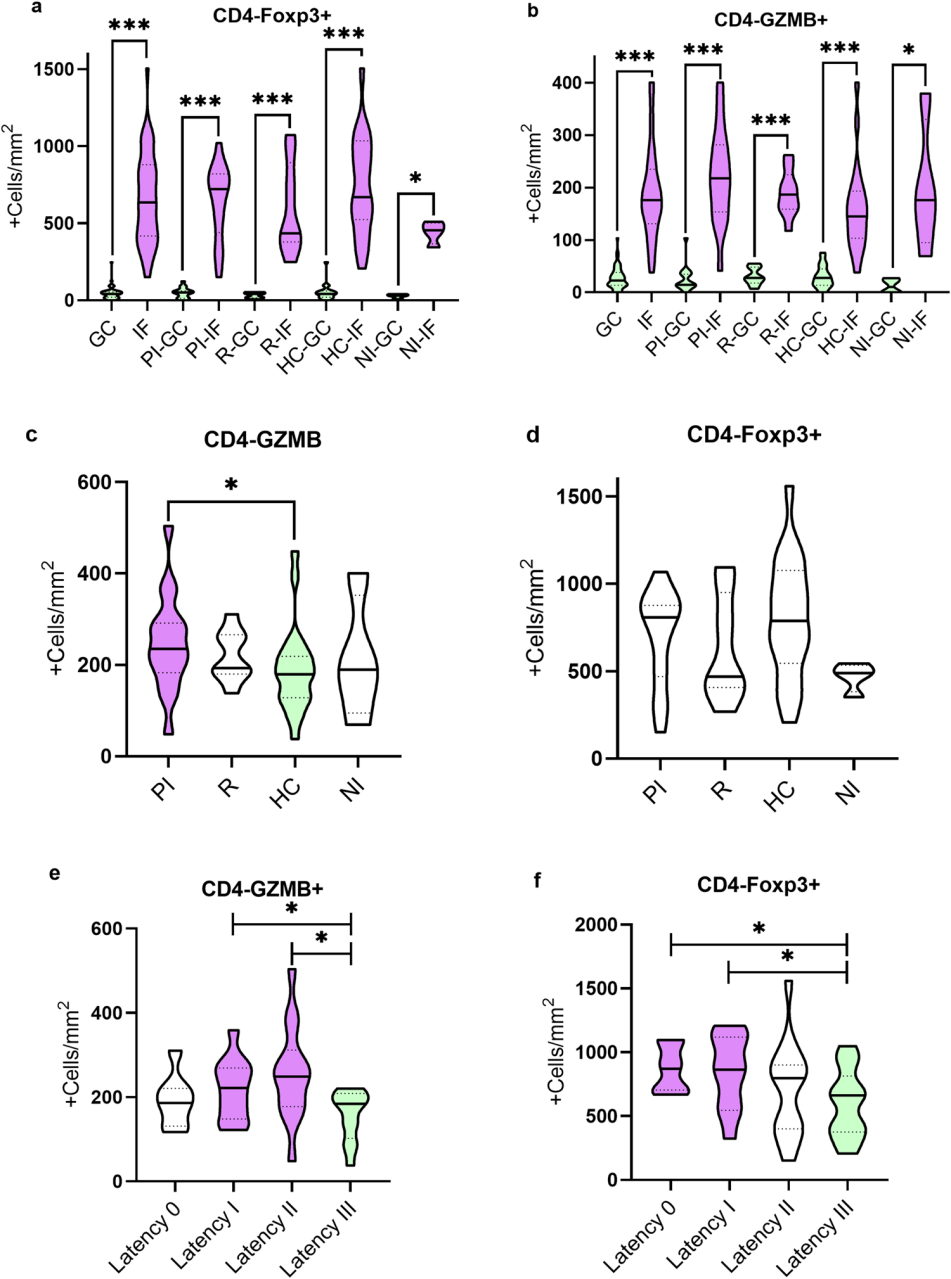
Histological distribution, patients classified according to EBV infection status and EBV latency profile with respect to CD4-Foxp3+ and CD4-GZMB+ expression. Histological distribution of CD4-Foxp3+ (**a**) and CD4-GZMB+ (**b**) expression at the germinal center (GC) and interfollicular region (IF) in entire cohort, primary infected (PI) patients, patients undergoing reactivation (R), healthy carriers (HC) and non-infected patients (NI). Patients classified according to EBV infection status with respect to CD4-GZMB+ (**c**) and CD4-Foxp3+ (**d**) expression in primary infected patients (PI), patients undergoing reactivation (R), healthy carriers (HC) and non-infected patients (NI) in the entire section. Patients classified according to EBV latency profile with respect to CD4-GZMB+ (**e**) and CD4-Foxp3+ (**f**) in patients with latency 0, I, II and III. ANOVA or Kruskal Wallis test, when appropriate, with Bonferroni or Dunn’s test for multiple comparison, respectively, where applied when the 4 infection groups or latency profiles were compared. Horizontal bars indicate M–W or T-test comparison, when appropriate, between 2 infection or latency profile groups. *p < 0.05 **p < 0.01 ***p < 0.001.

CD4-Foxp3+ and CD4-GZMB+ T cells counts were statically higher at IF region compared to GC in the entire cohort (*p*˂0.0001) and in each infection status analyzed separately (*p*˂0.05) (Fig. 3a, b).

Concerning EBV infection status, when the expression of these cells was compared in the different infection statuses, a significantly higher CD4-GZMB+ cell count was observed in PI patients compared to HC (*p* = 0.0370) (Fig. 3c). In contrast, no significant difference in CD4-Foxp3+ count was observed when comparing the different infection statuses (*p* > 0.05) (Fig. 3d) (Supplementary Table S4).

When patients were stratified according to EBV latency antigens expression in tonsils, CD4-GZMB+ cells count were significantly different between groups (*p* = 0.0372 ANOVA and *p* = 0.0293 Bonferroni III vs II). In fact, when paired analysis between groups was performed, the patients who presented latency III antigen displayed lower CD4-GZMB+ cells compared to those with latency profile II (*p* = 0.0128) and latency I (*p* = 0.0317) (Fig. 3e).

In addition, patients with latency profile III showed a lower CD4-Foxp3+ cell count compared to patients with latency profile 0 (*p* = 0.0272) and latency profile I (*p* = 0.0370) (Fig. 3f) (Supplementary Table S5). Concerning lytic antigen expression, neither CD4-Foxp3+ nor CD4-GZMB+ mean cell count showed statistical differences in cases that expressed lytic BMRF1 antigen vs those who did not express it (*p* > 0.05) (Supplementary Table S6).

### Correlation with IL-10 and TGF-β cytokines

Our group previously reported a regulatory environment in tonsils of children undergoing primary infection, where both IL-10+ and TGF-β+ expression prevailed^13^. Therefore, to investigate the potential association between those cytokines and the presence of CD4+ , Foxp3+ , GZMB+ , CD4-Foxp3+ and CD4-GZMB+ markers, positive cells counts in the tonsils of patients with distinct EBV infection statuses and viral latency profiles were correlated with IL10+ and TGF-β+ expression, evaluated by IHC.

Since latency III displayed lower GZMB+ cell count at the GC as well as CD4-GZMB+ cells in the entire cohort, correlation analysis between GZMB+ , CD4+ and CD4-GZMB+ cells count and IL-10+ and TGF-β+ cytokines was performed. A positive correlation between CD4+ and IL-10+ cells in the latency III group was observed (r = 0.553; *p* = 0.0498) (Supplementary Fig. 1A). Only GZMB+ cells negatively correlated with IL-10+ only in cases that expressed latency III antigens (*p* = 0.0128, r = − 0.6668) (Supplementary Fig. 1b), but unfortunately, this observation was not confirmed for CD4-GZMB+ expressing cells (*p* > 0.05). There was no correlation between CD4-GZMB+ with TGF-β+ and IL-10+ cells counts in the whole tonsil, GC or IF region in the entire patient cohort and within each infection status (*p* > 0.05).

Concerning the analysis of regulatory Foxp3+ marker, only R children exhibited a significant positive correlation between Foxp3+ and TGF-β+ cells (*p* = 0.0182, r = 0.7570) (Supplementary Fig. 2a). In line with the finding for Foxp3+ expressing cells, there was a positive correlation of CD4-Foxp3+ and TGF-β+ cells only on R patients (*p* = 0.0152, r = 0.7698) (Supplementary Fig. 2b). When IF and GC regions were discriminated, at the IF region, CD4-Foxp3+ and TGF-β+ cells displayed a statistical positive low correlation in the entire cohort (*p* = 0.0418, r = 0.292). Particularly in HC patients, a negative correlation between CD4-Foxp3+ and TGF-β+ cell counts specifically at the GC was demonstrated (*p* = 0.0201, r = − 0.5423) (Supplementary Fig. 2c). No correlation between CD4-Foxp3+ cell count with IL10+ and TGF-β+ within the remaining infection status and latency profiles were proved (*p* > 0.05).

Given that we previously demonstrated that lytic antigen prevailed in HC children^14^, when patients were discriminated according to the expression of BMRF1 lytic antigen we observed that the BMRF1-expressing patients had a positive correlation of Foxp3+ as well as CD4-Foxp3+ expressing cells with TGF-β+ cells in the entire tonsil (*p* = 0.0371, r = 0.6966 and *p* = 0.0366, r = 0.6979 respectively) (Supplementary Fig. 3a and b). In addition, only those cases that express BMRF1 lytic antigen showed a positive correlation between CD4-GZMB+ and TGF-β+ in the entire tonsil (*p* = 0.0138; r = 0.7766) (Supplementary Fig. 3c).

## Discussion

CD4+ T cells play an important role in immune-cell mediated control of gammaherpesvirus infection^15^. In EBV infection, the CD4 T cell response seems to target lytic cycle as well as latent antigens, even though no marked increase in total CD4 T cell counts was described in adults with acute IM. In contrast to the CD8 response, latent antigen-specific CD4 T cell responses tend to be larger than those directed against lytic antigens in primary symptomatic as well as in persistent infection^16^. This scenario does not mirror the situation of primary and persistent infections which occur asymptomatically in children from underdeveloped populations^3^, where little is known about CD4 T cell response.

Most studies concerning EBV immune response were performed on peripheral blood, but one of the most interesting anatomical sites to characterize viral infection is the tonsil, where EBV gets access to its target cell for primary infection and reactivation. In a preliminary analysis to understand the balance between EBV and the immune system to avoid malignant transformation, an increase of CD4 T cells around those cells that express viral LMP1 latent protein at the tonsillar GC was described in pediatric carriers^17^, suggesting the key role of CD4 immune response. In order to deepen those findings in different EBV-infection statuses as well as latency and lytic profiles, local CD4 T cells were evaluated in a pediatric series. Most CD4+ T cells were located outside the GC in the context of subclinical EBV infection in children, and no differences were found in PI patients in relation to other EBV infection statuses, in line with previous findings that described no marked increase in CD4 T cell response in IM^16^.

A key role of cytotoxic cells to restrict local EBV-infection in children with asymptomatic infection was suggested^17^. Nevertheless, this role might not be completely successful, since the recruitment of cytotoxic GZMB cells around EBV+ tumor cells in Hodgkin lymphoma^18,19^ and Diffuse large B cell lymphoma^20^ does not prevent lymphomagenesis. Furthermore, the presence of activated GZMB+ CD8+ T cells in EBV-associated gastric carcinoma were also described^21,22^. As expected for children from underdeveloped populations^23^, PI children displayed higher GZMB cell counts, possibly related to the asymptomatic infection observed in our series. We assume that GZMB+ cells could be NK and CD8 T cells, who are the main actors to kill infected and transformed cells through different mechanisms of target recognition and signaling cascades to achieve its goal^24^. However, since CD4+ T cells with cytotoxic activity were also described in response to chronic viral infections and as the antitumor response, their recruitment in different EBV infection status was explored. These cells are characterized by their ability to secrete GZMB and perforin to kill the target cells in an MHC class II-restricted fashion^25^. In fact, primary EBV infection in adults develops cytotoxic EBV-specific CD4 T cells, that secrete GZMB and perforin in response to ex vivo challenge with EBV-infected B cells, compared with adults HC^4,26^. In line with those findings, in our series local response in pediatric patients undergoing primary infection also induces higher numbers of CD4-GZMB cells compared to HC, suggesting a role of local cytotoxic CD4 T cells to successfully control EBV primary infection also in children. Furthermore, the killing activity by cytotoxic CD4 T cells was also proved against autologous lymphoblastoid cell lines (LCLs)^27^, as well as pre-endemic Burkitt lymphoma cells^28^, in consequence, further in vitro studies are required to explore if this specific cells in our pediatric population might be also able to restrict EBV-mediated transformation, and, ultimately, EBV-associated lymphomas.

Children with EBV-associated Burkitt or Hodgkin lymphoma showed an increased recruitment of Foxp3+ regulatory T cells at the tumor microenvironment^29,30^, but only associated with worse survival in Burkitt lymphoma^29^. In adult primary infection, peripheral CD4-Foxp3+ regulatory T cells are significantly lower compared with HC^31^, in particular these cells decrease after symptom onset^32^. In contrast, even though Foxp3+ cells count was higher in HC in our series, CD4-Foxp3+ cell counts displayed no differences among PI, HC and R patients, suggesting that Foxp3 in HC might not be expressed by CD4 T cells. A regulatory local environment expressing IL-10 and TGF-β was previously described in PI children^13^. Moreover, in adults with IM, IL-10 and TGF-β were also significantly raised compared to seropositive donors^31^. IL-10 could be expressed by regulatory T cells, since Foxp3-IL10+ regulatory Type 1 cells responding to EBNA-1 viral antigen were described^29^. In contrast, in this series only TGF-β expression correlated with both Foxp3+ and CD4-Foxp3+ regulatory T cells in children undergoing viral reactivation, indicating a collaborative role of regulatory T cells with TGF-β specifically in this particular population. Furthermore, IL-10 might not be related to regulatory T cells in this specific cohort.

Latent EBV antigens and the oncogenic potential of certain latent EBV proteins to trigger B cell transformation into immortalized proliferating LCLs has been extensively explored^33^. In addition, viral oncogenic potential in relation to immune response was proved. In fact, specific HLA class I alleles are strongly associated with EBV+ Hodgkin lymphoma and genetic polymorphisms of the NKG2D receptor gene are related with susceptibility to develop EBV-induced nasopharyngeal carcinoma^15^. Furthermore, defects related to T-cell functions, in genes such as SH2D1A, XIAP, ITK, MAGT1, CD27, and CD70, result in lymphoproliferation, and/or lymphoma^34,35^. CD4+ T cells can control the EBV-induced B-cell proliferation by the downregulation of the latency III promoter Cp and EBNA2 latent antigen, expressed exclusively on latency III pattern through soluble factors^5^. In this pediatric cohort, the lower GZMB and CD4-GZMB+ cells observed associated to the unrestricted latency III pattern may reveal an effective control of cytotoxic CD4 T cells to restrict viral latent antigen expression. CD4-Foxp3+ regulatory T cells could contribute to balance this response, as previously described in pediatric HL^19^, given that lower counts are also observed in less restricted latency III patterns. Since neither CD4-GZMB+ nor CD4-Foxp3+ correlate with IL-10+ cells, and, what’s more, GZMB+ negatively correlated with IL-10 expression in cases that expressed latency III antigens, this cytokine may be produced by other CD4 T cells rather than cytotoxic and regulatory CD4+ T cells. This delicate balance between CD4+ cytotoxic and regulatory response in the context of TGF-β immune regulation may be also observed in cases that express lytic antigens.

In summary, in this pediatric cohort cytotoxic CD4 T cells are involved in the control of EBV primary infection, as previously demonstrated for adults with IM^4,26^, probably related to the lack of symptoms in children. Moreover, these cells appear to play a role in effectively limiting the expression of latent viral antigens, as demonstrated in vitro^5^. Given that cytotoxic cells are important to mediate direct tumor cell killing^36^, and that in Argentina our group demonstrated a high incidence of EBV-associated lymphomas in children younger than 10 years old^10^, further studies are required to prove a potential failure in this specific cytotoxic response that could be related to an increased pediatric EBV-mediated lymphomagenesis.

## Materials and methods

### Patients and samples

Fifty pediatric patients aged between 1 and 15 years (mean and median 5) undergoing tonsillectomy due to non-reactive hyperplasia diagnosed according to international routine protocols for recurrent chronic inflammation at the Otorhinolaryngology Division, Ricardo Gutierrez Children’s Hospital (Buenos Aires, Argentina) were enrolled in this study. For the surgery to be performed, patients must be completely asymptomatic, without signs or symptoms of acute inflammation or infection such as fever, sore throat, or cough. Ethical Committee of the Ricardo Gutiérrez Children’s Hospital, approved the study protocol under the number CEI n° 20.37, and gave their supervision. All samples were collected after written informed consent (for patients older than 12 years old and legal guardians of children younger than 12 years old), and assent (7 to 12 years old patients and legal guardians of children older than 12 years old), following the national and international ethics standards and under the supervision of the Ethical Committee of the Ricardo Gutiérrez Children’s Hospital, in accordance to the Helsinki Declaration of 1975.

Formalin-fixed paraffin-embedded (FFPE) tonsil biopsy samples were obtained at the Pathology Division of Ricardo Gutierrez Children’s Hospital (Buenos Aires, Argentina).

### EBV infection status

At the time of surgery, a concomitant blood sample was collected from the patient to assess the serological EBV profile. Serology was performed by indirect immunofluorescence (IF) to determine the presence of serum antibodies: VCA-IgG, VCA-IgM, EA-IgG and EBNA1-IgG. Patients with primary infection (PI) were defined by the presence of VCA-IgM and VCA-IgG antibodies; healthy carrier patients (HC), by VCA-IgG and EBNA1-IgG presence; patients undergoing viral reactivation (R) by VCA-IgG, EA-IgG and EBNA1-IgG; and non-infected patients (NI) by the absence of EBV antibodies.

### EBERs in situ hybridization and immunohistochemistry for viral antigens

EBERs in situ hybridization (ISH) with ViewRNA ISH Tissue 1-Plex Assay (Affymetrix) was performed as described previously^14^. Immunohistochemistry (IHC) was performed in FFPE tonsil biopsy samples slices (3–4 μm). Primary antibodies for LMP1 (CS1-4 pool of clones, Dako) and EBNA2 (1E6 y R3 clones, Abcam) were used to evaluate viral latency antigens expression. Patients were classified into three latency profiles according to whether or not they expressed the following viral antigens: Latency 0 (no expression of viral antigens), Latency I (EBERS+), Latency II (EBERS+ and LMP1+) and Latency III (EBERS+ , LMP1+ and EBNA2+).

The primary antibody against BMRF1 (G3-E31 clone, Abcam) was used to evaluate the expression of this viral lytic antigen by IHC, as previously described^14^.

### Immunohistochemistry for cytokines expression: IL10 and TGF-β

The expression of IL-10+ and TGF-β+ was assessed to evaluate their association to CD4+ , Foxp3+ , GZMB+ , CD4-Foxp3+ and CD4-GZMB+. IL-10+ (clone Ab34843, Abcam) and TGF-β+ (clone Ab9758, Abcam) expression was evaluated by immunohistochemistry as previously described^13^.

### Double-staining for CD4 and GZMB/Foxp3

A double-staining was performed to assess the expression of Foxp3+ (clone 236A/E7, Thermofisher) and GZMB+ (clone GB11, Biorad) expressed in CD4+ (clone p35, Roche Ventana) T cells. The first step involved the labeling with the primary rabbit anti-CD4 antibody using the BenchMark XT automated equipment (Roche Ventana), with diaminobenzidine (DAB) as a chromogen for visualization. Then, after washing with TBS-Tween, mouse anti-Foxp3 or GRZMB antibodies were incubated for 1 h. Subsequently, second wash was performed and the slides were incubated with the secondary anti-mouse antibody labeled with Alexa Fluor 488 (Invitrogen), according to the manufacturer's instructions. Finally, staining with Hoescht nuclear stain was performed.

### Image acquisition and quantification

The tonsils were examined by focusing on two histological regions: the germinal center (GC) and the interfollicular (IF) regions. Ten representative images of each region were taken at 1000X, using ZEN 3.6 (blue edition) imaging platform, with A1 AxioScope (Carl Zeiss) microscope. Cell counts, image processing (appropriate adjustment of contrast and brightness) and channel fusion of the images were performed with Fiji software and the result was expressed as positive cells per unit area (+ cell/mm^2^)^37^.

### Statistical analysis

The data were analyzed using GraphPad Prism 8 software. Normality test was applied using Shapiro Wilks test. Comparison among groups was assessed by 1-way ANOVA or Kruskal–Wallis test according to the normality test results, followed by Bonferroni or Dunn’s test for multiple comparison, respectively, in case 1-way ANOVA or Kruskal–Wallis test were significant. Then, the mean comparison test was assessed by t-test or Mann–Whitney (MW) test between two groups, according to the normality test results. Correlations were assessed using Spearman or Pearson tests, when appropriate.

Outliers were defined using the Robust test to compare data median absolute deviation (Mad) in Excel. All tests were two-tailed, and *p* < 0.05 was considered statistically significant.

The results were graphically represented by violin plots, where the distribution of the data can be observed, and the median is represented as a solid line while the quartiles are represented as a dotted line.

### Supplementary Information

Supplementary Information.

## Data Availability

The datasets generated during and/or analyzed during the current study are available from the corresponding author on reasonable request.
