# Supplementary material for: Tonsillar cytotoxic CD4 T cells are involved in the control of EBV primary infection in children
Source: Sci Rep. 2024 Jan 25;14:2135. doi: 10.1038/s41598-024-52666-4 (PMC10810912; doi:10.1038/s41598-024-52666-4)
Supplement: Supplementary file 1 — Supplementary Information. [file 41598_2024_52666_MOESM1_ESM.pdf]

Tonsillar cytotoxic CD4 T cells are involved in the control of EBV primary infection in children

María Eugenia Amarillo, Agustina Moyano, Natalia Ferressini Gerpe, Elena De Matteo, Maria Victoria Preciado, Paola Chabay.

Infection status by EBV	VCA IgM	VCA IgG 1/10. 1/40. 1/320	EA IgG	EBNA1 IgG
Primary infection	+	+/-	+/-	-
Healthy carrier	-	+	-	+
Reactivation	-/+	+	+	+
Non-infected	-	-	-	-

Supplementary table S1. Infection status by EBV serological profile.

Latency profiles	Viral antigen expression
0	-
I	EBERs
II	LMP1. EBERs
III	EBNA2. EBERs. LMP1

Supplementary table S2. Latency profile by EBV protein expression

Infection status by EBV	Median age (range)
Primary infection	5 (2-12)
Healthy carrier	6 (2-15)
Reactivation	5 (2-11)
Non-infected	2 (1-11)

Supplementary table S3. Age distribution of EBV infection status

Infection status by EBV	N	CD4/mm² median (IQ range)			Foxp3/mm² median (IQ range)			GZMB/mm² median (IQ range)			CD4-Foxp3/mm² median (IQ range)			CD4-GZMB/mm² median (IQ range)		
		GC	IF	Total	GC	IF	Total	GC	IF	Total	GC	IF	Total	GC	IF	Total
Primary infection	16	**3063** (2253-3451)	**8232** (6911-10150)	**11129** (9791-13634)	**58.70** (13.81-117.4)	**752.7** (421.3-875.6)	**808.0** (497.2-973.7)	**145.0** (79.93-191.6)	**542.1** (429.9-725.1)	**721.7** (540.4-851.1)	**51.79** (13.81-62.15)	**721.7** (440.2-820.1)	**759.6** (433.3-871.9	**14.50** (13.81-34.53)	**217.5** (153.7-281.4)	**234.8** (183.0-291.1)
Healthy carrier	21	**2866** (2431-3992)	**8591** (6985-9865)	**11325** (10317-13228)	**48.34** (20.72-107.0)	**669.9** (576.6-1091)	**863.2** (645.7-1205)	**110.5** (54.56-200.3)	**435.1** (313.9-580.1)	**550.4** (424.7-688.5)	**44.89** (20.72-62.15)	**669.9** (531.7-1036)	**787.3** (604.3-1088)	**27.62** (13.81-44.89)	**145.0** (103.2-193.4)	**179.5** (128.1-218.2)
Reactivation	9	**2592** (2565-3384)	**8100** (6426-8739)	**10642** (9202-12161)	**34.53** (20.72-46.61)	**442.0** (379.8-932.3)	**469.6** (410.9-1087)	**124.3** (100.1-172.6)	**429.5** (317.7-628.4)	**649.1** (419.2-812.1)	**41.43** (17.26-51.79)	**435.1** (379.8-894.3)	**469.6** (407.4-949.5)	**27.62** (17.75-48.34)	**186.5** (158.8-224.4)	**193.4** (180.0-265.9)
Non-infected	4	**3391** (3128-4161)	**7571** (6507-10734)	**11535** (9922-14035)	**31.08** (12.09-39.71)	**455.4** (367.7-502.2)	**489.9** (383.3-535.0)	**55.25** (19.51-65.60)	**300.4** (253.8-590.4)	**362.6** (276.7-645.7)	**31.08** (12.09-39.71)	**455.4** (367.7-502.2)	**489.9** (383.3-535.0)	**10.36** (0.000-25.90)	**176.1** (94.95-329.7)	**189.9** (94.95-352.2)

Supplementary table S4. Median and Interquartile (IQ) range according to EBV infection status. GC: germinal center; IF: interfollicular region.

EBV Latency profile	N	CD4/mm² median (IQ range)			Foxp3/mm² median (IQ range)			GZMB/mm² median (IQ range)			CD4-Foxp3/mm² median (IQ range)			CD4-GZMB/mm² median (IQ range)		
		GC	IF	Total	GC	IF	Total	GC	IF	Total	GC	IF	Total	GC	IF	Total
0	7	**2728** (2445-3529)	**9316** (8826-10241)	**12707** (11760-13729)	**48.34** (20.72-103.6)	**966.8** (738.9-1084)	**987.5** (787.2-1146)	**78.73** (41.43-207.2)	**448.9** (400.5-614.6)	**566.3** (435.1-711.3)	**48.34** (20.72-103.6)	**745.8** (656.0-1036)	**870.1** (704.4-1095)	**13.81** (6.906-48.34)	**158.8** (124.3-207.2)	**186.5** (131.2-221.0)
I	12	**2679** (2327-3190)	**8100** (7921-10676)	**10642** (10324-14509)	**55.25** (34.53-96.68)	**875.6** (607.7-1153)	**972.3** (628.4-1264)	**186.5** (118.8-226.2)	**488.2** (367.2-623.2)	**657.4** (500.8-839.0)	**55.25** (27.62-89.77)	**794.2** (524.8-1057)	**864.6** (545.6-1119)	**34.53** (27.62-34.53)	**181.3** (116.5-240.0)	**221.7** (148.5-269.3)
II	14	**2842** (2374-3373)	**7804** (6075-9483)	**10977** (8772-12507)	**48.34** (13.81-75.96)	**587.4** (366.0-820.1)	**794.2** (386.7-904.7)	**113.9** (65.60-202.0)	**504.1** (324.6-711.3)	**656.0** (481.7-799.3)	**43.85** (20.72-54.38)	**483.4** (352.2-783.8)	**787.3** (386.7-866.7)	**17.95** (13.81-50.07)	**217.5** (150.2-293.5)	**249.0** (177.8-311.8)
III	13	**3232** (2439-3719)	**7603** (6056-8649)	**10534** (9029-12403)	**41.43** (13.81-200.3)	**607.7** (366.0-766.5)	**663.0** (379.8-991.0)	**107.0** (86.32-136.4)	**442.0** (265.9-663.0)	**550.4** (386.7-787.3)	**41.43** (10.36-55.25)	**607.7** (366.0-759.6)	**663.0** (376.4-814.9)	**20.72** (10.36-87.62)	**158.8** (91.50-183)	**184.0** (102.4-208.9)

Supplementary table S5. Median and Interquartile (IQ) range according to latency profiles. GC: germinal center; IF: interfollicular region.

BMRF1 expression	N	CD4/mm² median (IQ range)			Foxp3/mm² median (IQ range)			GZMB/mm² median (IQ range)			CD4-Foxp3/mm² median (IQ range)			CD4-GZMB/mm² median (IQ range)		
		GC	IF	Total	GC	IF	Total	GC	IF	Total	GC	IF	Total	GC	IF	Total
BMRF1+	10	**3049** (2466-3617)	**7728** (6424-8836)	**10612** (9654-11483)	**48.34** (17.26-145.0)	**642.2** (417.8-915.0)	**745.8** (424.7-1042)	**96.68** (65.60-134.7)	**421.3** (272.8-490.3)	**490.3** (386.7-574.9)	**41.43** (13.81-55.25)	**635.3** (412.6-911.6)	**742.4** (419.5-961.6)	**20.72** (13.81-27.62)	**165.7** (155.4-202.0)	**189.9** (177.8-215.8)
BMRF1-	40	**3004** (2445-3487)	**8524** (6733-9875)	**11506** (9951-13231)	**44.89** (20.72-96.68)	**635.3** (421.3-918.5)	**752.7** (459.2-1126)	**113.9** (69.06-191.6)	**455.8** (333.0-664.7)	**625.0** (457.0-787.3)	**41.43** (20.72-56.97)	**621.5** (414.3-870.1)	**687.1** (459.2-935.7)	**27.62** (13.81-41.43)	**177.8** (124.3-252.1)	**210.6** (138.1-279.7)

Supplementary table S6. Median and Interquartile (IQ) range according to BMRF1 expression. GC: germinal center; IF: interfollicular region.

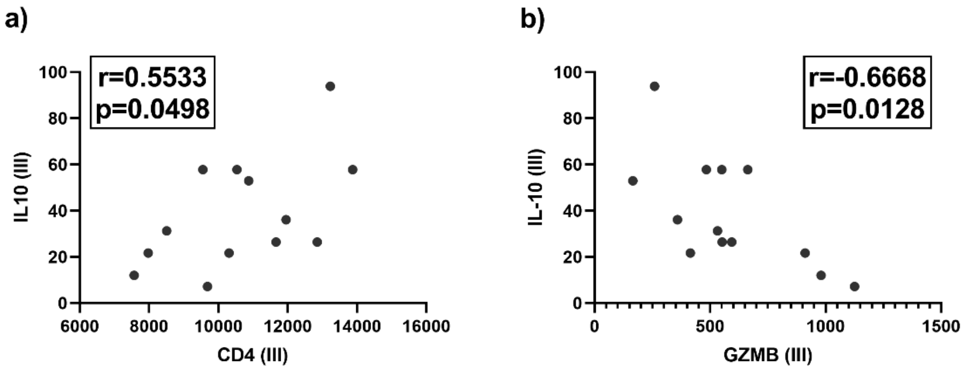

Supplementary figure 1. Correlation of IL-10+ cells with CD4 (a) and GZMB (b) in latency III.

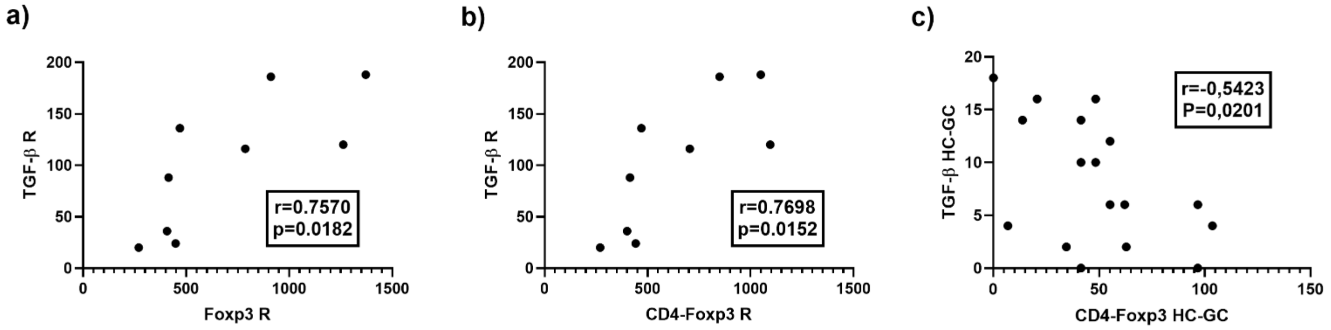

Supplementary figure 2. Correlation of TGFβ+ cells with Foxp3 (a) and CD4-Foxp3 (b) in reactivation, and with CD4-Foxp3 at the GC (c).

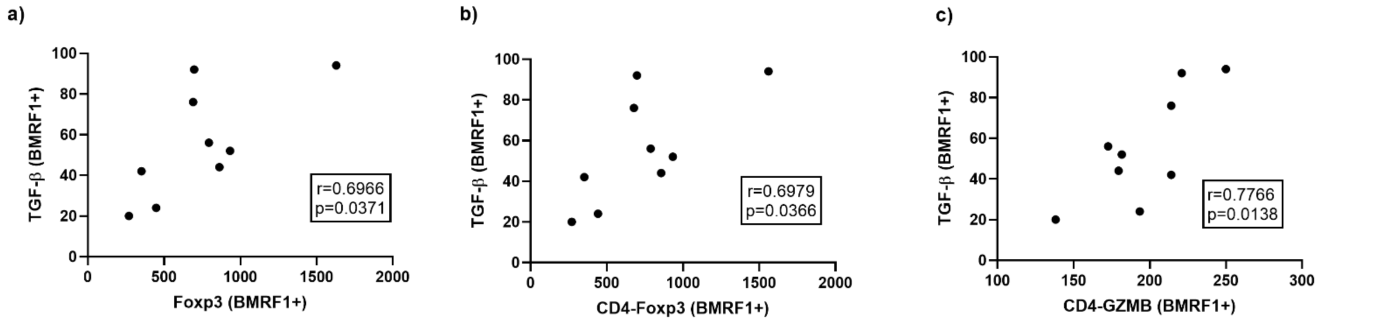

Supplementary figure 3. Correlation of TGFβ+ cells with Foxp3 (a), CD4-Foxp3 (b) and with CD4-GZMB (c) in BMRF1+ cases.
